# Modeling physiological and pathological human neurogenesis in the dish

**DOI:** 10.3389/fnins.2014.00183

**Published:** 2014-07-24

**Authors:** Vania Broccoli, Serena G. Giannelli, Pietro G. Mazzara

**Affiliations:** Stem Cells and Neurogenesis Unit, Division of Neuroscience, San Raffaele Scientific InstituteMilan, Italy

**Keywords:** ESCs, iPSCs, neural tube, human neurogenesis, rosettes, neural crest, *in vitro* disease modeling, self-aggregation

## Abstract

New advances in directing the neuronal differentiation of human embryonic and induced pluripotent stem cells (hPSCs, abbreviation intended to convey both categories of pluripotent stem cells) have promoted the development of culture systems capable of modeling early neurogenesis and neural specification at some of their critical milestones. The hPSC-derived neural rosette can be considered the *in vitro* counterpart of the developing neural tube, since both structures share a virtually equivalent architecture and related functional properties. Epigenetic stimulation methods can modulate the identity of the rosette neural progenitors in order to generate authentic neuronal subtypes, as well as a full spectrum of neural crest derivatives. The intrinsic capacity of induced pluripotent cell-derived neural tissue to self-organize has become fully apparent with the emergence of innovative *in vitro* systems that are able to shape the neuronal differentiation of hPSCs into organized tissues that develop in three dimensions. However, significant hurdles remain that must be completely solved in order to facilitate the use of hPSCs in modeling (e.g., late-onset disorders) or in building therapeutic strategies for cell replacement. In this direction, new procedures have been established to promote the maturation and functionality of hPSC-derived neurons. Meanwhile, new methods to accelerate the aging of *in vitro* differentiating cells are still in development. hPSC-based technology has matured enough to offer a significant and reliable model system for early and late neurogenesis that could be extremely informative for the study of the physiological and pathological events that occur during this process. Thus, full exploitation of this cellular system can provide a better understanding of the physiological events that shape human brain structures, as well as a solid platform to investigate the pathological mechanisms at the root of human diseases.

## Using hPSC-derived rosette formation to model early neurulation processes

The unique developmental potential and replicative capacity of hPSCs offers a nearly unlimited source of specific somatic cell types that can be exploited for *in vitro* mechanistic studies or cell transplantation therapies. Remarkably, neuronal cells were among the first lineages to be differentiated using hPSCs (Reubinoff et al., 2001; Zhang et al., 2001). Neuronal induction was first obtained by promoting the differentiation of hPSCs in aggregate-like embryoid bodies. Subsequently, aggregates were placed in stringent serum-free culture conditions, which selectively facilitate the survival and growth of neural cells. This transition toward the neural lineage is readily manifested in hPSCs (but not in their murine counterparts) because of the appearance of rosette-like structures within the differentiating hPSC colonies (Reubinoff et al., 2001; Zhang et al., 2001). These structures develop from progenitor cells, which line up close together to form a round, columnar epithelium that is reminiscent of blooming rosettes when viewed under bright light.

**Figure 1:**
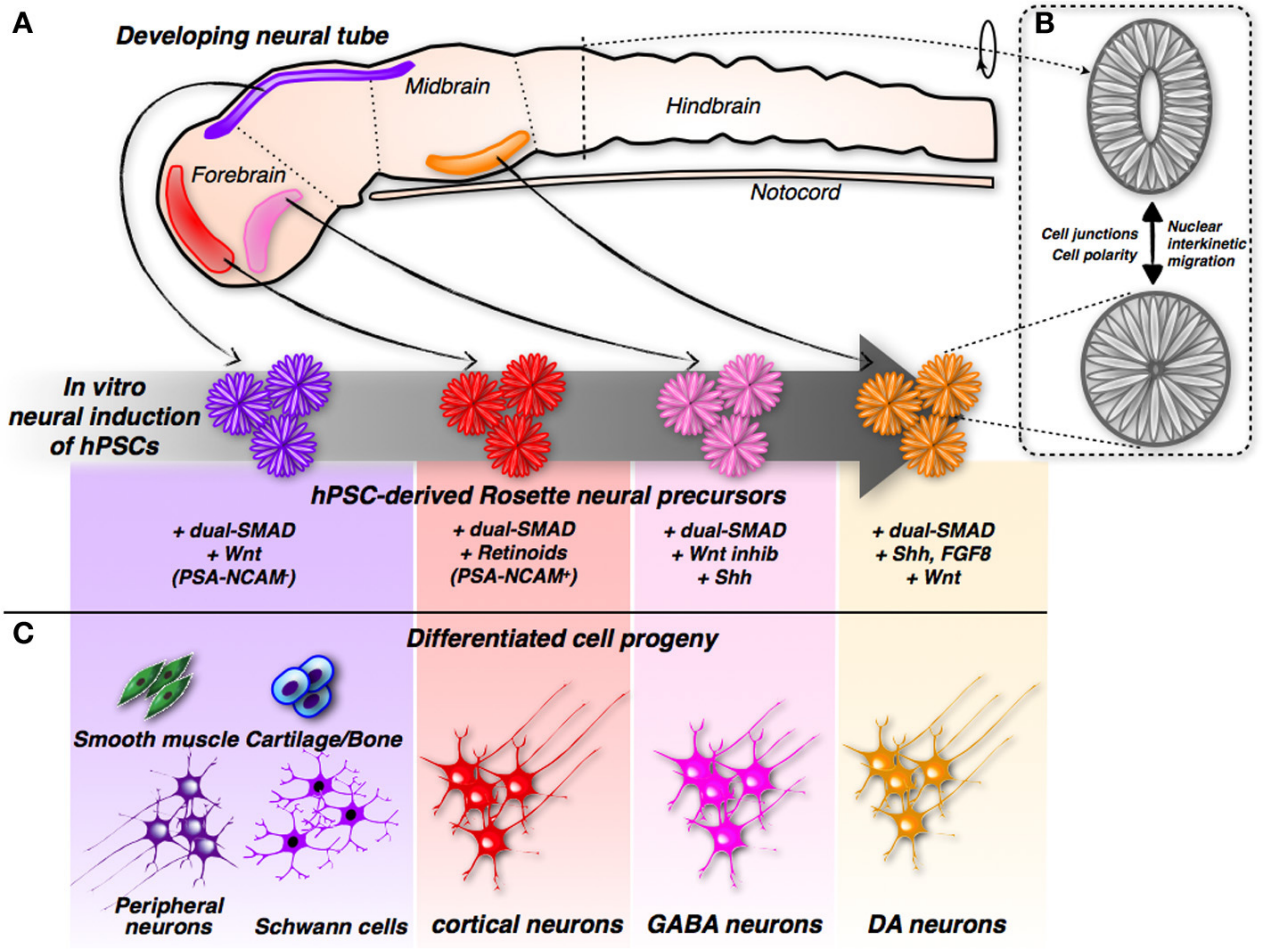
**Induction and differentiation of iPSC-derived rosette neural progenitors. (A)** Modulation of neurodevelopmental molecular pathways by different combinations of small molecules can direct hPSCs into rosette neural progenitors with different positional identities along the developing neural tube. **(B)** The hPSCs-derived rosette and the neural tube share the same spatial organization showing cells with a remarkable polarization and cell-junction compartmentalization. In addition, relative nuclei position is depending by the cell-cycle stage in both systems. **(C)** According to their specific early commit-ment, hPSCs-derived rosette neural progenitors generate distinct neuronal sub-types and, in the case of hNCPCs, even non-neuronal somatic cells.

Further examination of these structures revealed that the rosettes are formed from neural progenitors. These neural progenitors are endowed with highly polarized morphology, as indicated by the presence of tight and adherence junctions at the side facing the internal lumen, while the external side is strongly enriched in laminin-rich extracellular matrix (Lazzari et al., 2006; Elkabetz et al., 2008; Colleoni et al., 2010). This architecture recapitulates the cellular organization of the neural tube, the embryonic primordium of the entire central nervous system (CNS), in both shape and function. In fact, within rosettes, the nuclei of neural progenitor cells undergo a stereotyped movement known as interkinetic nuclear migration, which is harmonized to the cell cycle stage and specific to authentic neural tube cells (Taverna and Huttner, 2010). Nuclei undergoing DNA synthesis localize at the outer edge of the neural tube, while mitotic divisions are confined to its innermost core, a pattern replicated in the same sequence by nuclei within the rosettes (Lazzari et al., 2006; Elkabetz et al., 2008; Colleoni et al., 2010). Therefore, rosettes share the same elementary organization of the developing neural tube; hence, they are equivalent to the developing neural tube with respect to structure and function (Figure 1). Rosette neural progenitors intertwine to create overlapping cellular layers; however, they remain constrained to the surface where they anchor. How rosettes can be adapted to the three-dimensional (3D) space, and which morphology and growth pattern they will follow in these culture conditions, have not yet been ascertained. This setting might enable the *in vitro* reproduction not only of neural tube organization, but also (and even more challenging) its organogenesis.

During development, the neural tube is highly patterned along the anterior-posterior and ventral-dorsal axes (Muñoz-Sanjuán and Brivanlou, 2002). In particular, its anterior portion is invariably committed to give rise to the different brain regions. It is worth noting that neural rosettes, when cultured in the mentioned culture conditions, acquire a specific regional code that coincides with the anterior-dorsal section of the developing neural tube, as demonstrated by the acquisition of a specific set of regional molecular markers (Elkabetz et al., 2008; Colleoni et al., 2010; Hu et al., 2010) (Figure 1). Considering that rosettes are induced in a medium that lacks any molecular inducer, these findings suggest that the commitment of the anterior-dorsal region represents the default state during neuronal induction, and only subsequent inductive clues, either cell or non-cell autonomous, might override this basal state, imposing other regional identities.

## From neural tube-like rosettes to neural crest cell progenitors

In serum-free neurobasal culture conditions, each differentiating hPSC rosette is scattered in an array of disorganized and unpatterned cells. Remarkably, the expression of two specific markers (p75 and HNK1) identified many of these cells as neural crest cells (Lazzari et al., 2006; Gossrau et al., 2007; Lee et al., 2007, 2010). Molecular analyses corroborated the acquisition of the neural crest identity, as shown by expression of the associated molecular determinants. Temporal analysis illustrated how neural crest cells originated by delaminating from rosette structures in a process similar to the epithelial-mesenchymal transition (EMT), which normally occurs during development when nascent neural crest cells arise from the neural tube (Curchoe et al., 2010). Spontaneous emergence of the neural crest component *in vitro* provides additional evidence that PSC-derived rosette structures acquire dorsal identity, since this is the side of the neural tube from which the neural crest is selectively induced during development (Le Douarin et al., 2004).

In agreement with this view, treatment of rosettes with Sonic hedgehog (Shh), a ventralizing morphogen expressed by the floor plate of the ventral neural tube, inhibits the expression of neural crest-specific genes while activating ventral neural tube markers (Colleoni et al., 2010). More importantly, subsequent culturing of neuralized hPSC colonies that had been exposed to epigenetic stimulation with the growth factors bFGF and EGF enabled the generation of stable cell lines of human neural crest progenitor cells (hNCPCs) (Lazzari et al., 2006; Lee et al., 2007, 2010; Colleoni et al., 2010). hPSC-derived hNCPCs exhibited a stable phenotype, even after sustained proliferation *in vitro* and multiple single cell disaggregation passages.

Notably, although hPSC colonies initially included CNS-like rosette progenitors, the rosette progenitors stopped multiplying and died out over few passages, giving rise to a population of pure hNCPCs (Colleoni et al., 2010). This probably occurred because rosette neural progenitors are extensively polarized epithelial cells, and therefore are susceptible to cell death upon single cell dissociation. Conversely, hNCPCs retained an intrinsic resilience to cell death that might have been gained with the EMT process and the acquirement of mesenchymal-like cell properties (Vega et al., 2004; Robson et al., 2006). Interestingly, the withdrawal of mitogens induced cell cycle exit and the differentiation of neural crest progenitors into an array of somatic cells, including sympathetic and sensory neurons, glial cells, melanocytes, myofibroblasts, cartilage, and bone cells (Lee et al., 2007; Colleoni et al., 2010). Although neural lineages are well represented, their *in vitro* maturation is a weeks-long process. In addition, glial cells progressed to a Schwann cell progenitor stage; these progenitors were positive for S100β and GFAP, although no sign of occurring myelination was ever detected (Lee et al., 2007; Colleoni et al., 2010). Therefore, it remains unknown whether hPSC-derived Schwann cells can be induced to differentiate, or whether they are able to generate functional myelin sheaths around axonal tracts. Clarification of this point will enable the establishment of a valuable model of human myelination.

Furthermore, taking into consideration other neural crest derivatives, an interesting study has recently determined the experimental conditions necessary for the maturation and enrichment of melanocytes from differentiating hNCPCs (Mica et al., 2013). Finally, mesodermal derivatives, including cartilage and bone, have been equally generated from hNCPCs (Lee et al., 2007; Colleoni et al., 2010). This finding may be of great interest for regenerative therapies, exploiting bone and cartilage replacement to treat, for example, trauma or osteoporosis. However, to continue along this direction, it is necessary to address the quality and type of cartilage that hNCPCs are able to generate, as well as (even more importantly) its stability, robustness, and longevity after *in vivo* transplantation.

The induction of both neural and mesenchymal cell lineages indicates that hPSC- derived NCPCs acquire a cranial identity in culture, because only head neural crest contributes to mesoderm-derived structures in the embryo (Le Douarin et al., 2004). Remarkably, clonal analysis identified hNCPCs with mesodermal and/or ectodermal potential similar to what has been described regarding hPSC-derived neural crest cells (Baroffio et al., 1991; Lee et al., 2007). However, different culture conditions or durations in culture can strongly drive hNCPCs from a neural to a mesenchymal phenotype (Lee et al., 2007; our unpublished results). Indeed, the exposure of hNCPCs to a serum-containing medium upregulated mesenchymal markers (e.g., CD73) and stimulated their differentiation into mesodermal derivatives (e.g., smooth muscle, cartilage, and bone cells) (Lee et al., 2007). Therefore, hNCPCs represent an ideal cellular system in which to investigate the molecular mechanisms that regulate this dual cell fate potential, as well as their subsequent final differentiation into a variety of distinct cell types.

## Long-lasting neuronal progenitors cells (hNPCs)

While isolating and expanding hNCPCs from hPSC-derived neural rosettes is a straightforward process, the generation of hNPCs is unfortunately hampered by their low survival rate upon rosette dissociation (Elkabetz et al., 2008; Koch et al., 2009). However, this hurdle has been significantly attenuated by the introduction of the Rho-associated protein kinase (ROCK) inhibitor Y-27632, which inhibits single-cell-induced death (Watanabe et al., 2007). hNPCs, which are capable of extensive self-renewal, clonogenicity, and multipotency, have been derived from rosettes by generating neurosphere-like floating aggregates or monolayer cultures (Zhang et al., 2001; Conti et al., 2005). The purity of hNPC cultures has yet to be convincingly assessed, and might be jeopardized by the presence of contaminating hNCPCs. This is an important caveat because many protocols rely on the manual isolation of rosettes, and therefore likely permit the incorporation of closely abutting hNCPCs, which can easily outnumber the hNPCs owing to their better survival and growth rate in the same culture conditions. Taking all of this into consideration, it is always worthwhile to test the presence of p75+ cells in hNPC cultures in order to verify neural crest cell contaminants. Kim et al. (2012) have conceived a valuable approach to prevent such contamination; they demonstrated that polysialylated-neural cell adhesion molecule is strongly enriched in rosette hNPCs, and thus cell sorting for this epitope enables the isolation of a pure population, devoid of any cellular contaminants.

The polysialylated-neural cell adhesion molecule-negative cell fraction is mainly comprised of hNCPCs, thus providing an approach to purify both cells types in a single-step procedure (Kim et al., 2012). Moreover, long-lasting proliferative hNPCs represent a renewable source of neurons and glia that, once established, is independent of hPSC culture. However, growth factor-dependent hNPC expansion might also lead to undesired effects. Indeed, Bilican et al. (2014) noted that chronic exposure to EGF might cause NPCs to lose their anterior identity while assuming a hindbrain regional code. Interestingly, the expansion of hNPCs in the presence of bFGF and physiological levels (3%) of O_2_, is sufficient to enable long-term proliferation while maintaining the anterior character that is fundamental for stable differentiation of committed cortical neurons. How hNPCs are able to modify their competent state with respect to regional specification and give rise to different neuronal subtypes is still unclear. Some reports suggest that early hNPCs might still respond to inductive signals in order to acquire a different regional identity (Koch et al., 2009); however, they are unlikely to be able to maintain this capability without change over time and after extensive cell passaging. Given these last observations, several procedures have been established with the aim of generating a single neuronal subtype using direct differentiation of hPSC-derived neural rosettes, some of which are described in the next Section.

## Cortical neurons

Although neural rosettes exhibit a default anterior regional code, their dissociation and subsequent neuronal differentiation in standard culture conditions (e.g., absence of morphogen supplementation) leads to a population of mature neurons that are representative of various brain regions. However, prolonged exposure to the BMP inhibitor Noggin is sufficient to efficiently induce differentiation mainly of cortical neurons (Espuny-Camacho et al., 2013) (Figure 1). In addition, the presence of retinoids (retinol-acetate and all-trans retinol) during neural induction and differentiation maximizes the conversion of cortical neurons (Shi et al., 2012). Both procedures converge on the progression of cortical neuronal differentiation with the initial generation of deep layer neurons; superficial layer neurons are generated later, during the development of the cerebral cortex. Therefore, the temporal sequence of neuronal subtype specification in this system mirrors the *in vivo* occurrence of the same process (Greig et al., 2013). Based on these data, one can envisage that this pattern of *in vitro* neurogenesis is mainly mediated by cell autonomous mechanisms, which might be intrinsically encoded within the neuronal progenitors. The generation of both deep and upper cortical neurons from hPSCs covers a very extensive timeframe, which might last for up to 3 months of culture, which dovetails with the delay in cortical neurogenesis that occurs in human embryos compared with mouse embryos (Shi et al., 2012; Espuny-Camacho et al., 2013). More importantly, hPSC-derived cortical neurons grafted into the frontal cortex of the neonatal brain were able to survive, mature, and extend their axons to the proper neuronal targets according to their layer specification (Espuny-Camacho et al., 2013). These data indicate that the use of this *in vitro* system enables the full acquisition of subtype identity, which is then fully elaborated after transplantation. Finally, the grafted human neurons developed functional properties and synaptic connectivity within 3 months after transplantation. It would be of great interest to challenge the transplantation of these neurons in healthy and injured adult brain, in order to verify whether they are able to mature and functionally integrate even in more prohibitive conditions.

## GABAergic neurons

In contrast to excitatory (glutamatergic) cortical pyramidal neurons that project long distance, inhibitory (GABAergic) cortical interneurons make local synapses and are essential for the maintenance of balanced activity within neural circuits (Marín, 2013; Kepecs and Fishell, 2014). Interneuronal dysfunctions have been described in several disorders, including epilepsy, autism, schizophrenia, and Tourette's syndrome (Valiente and Marín, 2010; Alvarez Dolado and Broccoli, 2011; Marín, 2012). The direct differentiation of hPSCs into telencephalic GABAergic neurons has been achieved recently (Maroof et al., 2013; Nicholas et al., 2013). In both studies, early telencephalic GABAergic commitment of hPSCs was reported by the expression of green fluorescent protein (GFP) under the control of the Nkx2.1 promoter, which is fundamental for setting up the appropriate conditions for the specification of this neuronal subtype. In fact, optimal conditions were attained by combining exposure to BMP and Wnt inhibitors, which prime telencephalic identity, with Shh signaling stimulation, which induces ventral neuronal character, thus reaching up to 70% of Nkx2.1-GFP-positive, hPSC-derived, GABAergic neuronal precursors (Figure 1). Cortical interneurons include a different set of neuron types that have distinct roles in cortical development and function (Marín, 2013; Kepecs and Fishell, 2014). In these conditions, the prevalence of somatostatin-positive cells was observed, while many fewer interneurons expressing parvalbumin or VIP were detected. Thus, more work is needed to identify permissive conditions for the effective derivation of the later subtypes. Co-cultures with astrocytes or hippocampal neurons promoted hPSC-derived GABAergic neuronal maturation and functionality, including repetitive action potential firing, ionic channel currents, and synaptic currents. To directly prove their inhibitory synaptic activity, GABAergic neurons were excited by an optogenetic protocol that evoked robust postsynaptic currents in neighboring neurons, which were sensitive to the GABA antagonist bicuculline (Nicholas et al., 2013). Thus, hPSCs can be induced to differentiate into functional inhibitory GABAergic interneurons. It is interesting to point out that upon transplantation into neonatal brains, a fraction of the grafted neurons matured in the host neural tissue, gaining functional properties and synaptic connectivity, although several months of engraftment were required (Nicholas et al., 2013). These last results are particularly compelling in light of the fact that the transplantation of mouse native GABAergic precursors has proven therapeutic in treating animal models of epilepsy, schizophrenia, and Parkinson's disease (PD) (Southwell et al., 2014; Tyson and Anderson, 2014). All of these different pathological conditions have benefited from GABAergic neuronal transplantation, likely because it favors the re-organization of the neuronal circuit connections and the re-establishment of the correct balance between excitation and inhibition in cortical circuits (Southwell et al., 2014; Tyson and Anderson, 2014). In particular, this approach might have a straightforward application regarding the many forms of epilepsy that eventually reach an irreversible, drug-resistant stage.

## Dopaminergic neurons

Substantia nigra (A9) midbrain dopaminergic neurons are specifically lost during the initial phase of PD. The consequent loss of dopamine availability in striatal tissue is responsible for the troublesome motor impairments that severely affect PD patients. In fact, most of the drugs used to treat PD aim simply to restore sufficient levels of dopamine in the striatum, but cannot reverse or block the loss of dopaminergic neurons (Meissner et al., 2011). In this context, dopamine neuron replacement has been envisioned as a potential therapeutic strategy during the last decades (Dunnett et al., 2001; Lindvall and Björklund, 2011).

Transplantation of midbrain human fetal tissue provided a solid basis for a cellular approach to PD, confirming the ability of transplanted neurons to integrate into the host striatal tissues and sustain functional integration and a therapeutic effect (Barker et al., 2013). However, this approach had little clinical benefit, mainly because of the broad cell heterogeneity of the transplanted tissue, its minimal availability, and scarce immuno-compatibility (Lindvall and Björklund, 2004; Barker et al., 2013). Even worse, these issues were responsible for the development of the troubling side effect of graft-induced dyskinesia, which is experienced by some of the patients (Lane et al., 2010).

Given this context, hPSCs have long been considered a better renewable source of dopamine neurons that could potentially overcome these hurdles. To this purpose, many protocols have been developed for the *in vitro* differentiation of hPSCs into this particular class of neurons. Combined exposure to the morphogens Shh and FGF8, known to contribute to ventral midbrain neural commitment in the developing neural tube, was also found to instruct mouse and human PSCs to differentiate into tyrosine hydroxylase (TH)-positive dopamine neurons (Kim et al., 2002; Perrier et al., 2004; Lee et al., 2009) (Figure 1). However, the brain contains distinct dopamine neuronal clusters that differ with regard to function, connectivity, and molecular marker expression. This is a crucial issue because only transplantation of A9 [and not, for example, VTA (A10)] resident dopamine neurons can achieve maximal behavioral recovery in a mouse model of PD (Grealish et al., 2010). Reasonable graft-induced recovery depends on the correct innervation (and thereby the functional activation) of the striatum, which represents the physiological target structure of A9, but not A10, dopamine neurons (Grealish et al., 2010).

These results have far-reaching implications, indicating that the therapeutic benefits of cell transplantation approaches depend on the replacement of native neurons with cells that are close, if not identical, in nature and identity. Improvements in the differentiation of hPSCs into fully committed midbrain dopamine neurons have been attained by two recent studies (Kriks et al., 2011; Kirkeby et al., 2012). Both employed sustained stimulation of Wnt signaling (by inhibiting the enzyme glycogen synthase kinase 3β, GSK-3β) to robustly induce the differentiation of hPSCs into dopamine neurons (Figure 1). Neurons derived using this protocol exhibited midbrain character, as confirmed by the expression of the regional markers FoxA2 and Lmx1a (Kriks et al., 2011; Kirkeby et al., 2012). Remarkably, transplantation of these midbrain-specific dopamine neurons in the striatum resulted in significant behavioral recovery in both rodent and primate models of PD (Kriks et al., 2011; Kirkeby et al., 2012).

This transplantation strategy is designed to allocate transplanted dopamine neurons close to their physiological target (striatal neurons) in order to facilitate their functional connectivity, although grafted neurons would be ectopically located regarding their natural midbrain position. It is unknown whether this new neural circuit, its components necessarily re-organized, can fully restore physiological function without any concomitant behavioral alterations. To overcome this limitation, it would be interesting to test whether hPSC-derived neurons transplanted in the substantia nigra are able to re-establish nigro-striatal connections. Although the adult brain parenchyma is minimally permissive to axonal regrowth, grafted fetal dopaminergic neural precursors are surprisingly capable of generating a nigro-striatal pathway with an outgrowth pattern that matches the anatomy of the intrinsic system (Thompson et al., 2009). These results reveal some opportunities to reconstruct native neuronal circuits using a cell transplantation approach in the adult brain. Whether a similar approach can be envisioned using hPSC-derived dopamine neurons remains to be demonstrated.

## *In vitro* neuronal maturation and aging

*In vitro* differentiation of hPSCs into neurons is a long and multistep process that matches all of the stages of embryonic neuronal commitment and differentiation. Therefore, the neurons that result from hPSC induction appear to be very immature soon after their specification; they acquire functional properties only after several weeks in culture. Mature functional and synaptic properties are observed in the majority of the hPSC-derived neurons after 8–12 weeks of culture (Ricciardi et al., 2012; Verpelli et al., 2013). Thus, this delay in functional maturation of the hPSC-derived neurons *in vitro* certainly hampers the conduct of functional studies using this system.

Supplementation of the culture medium with neurotrophins and cAMP can enhance neuronal differentiation and restrain cell death events (Soldner et al., 2009). However, a major differentiating stimulus is provided when hPSC-derived neurons are co-cultured on a primary astrocyte feeder layer. In fact, neuronal differentiation, dendrite development, excitability, and synaptic activity are all accelerated from 8- to 20-fold when hPSC-derived neurons are cultured on astrocytes rather than on laminin (Tang et al., 2013). Zhang et al. (2013) recently devised an interesting alternative. They have shown that hPSC differentiation can yield a high level of functional glutamatergic neuronal cells in less than 2 weeks through the forced expression of only one transcription factor, Neurogenin 2. This is an attractive procedure because neurons are generated directly from naïve hPSCs in a single manipulation step, thereby skipping all of the intermediate steps and saving time and labor. A similar approach has been employed recently to accelerate the generation of functional dopamine neurons by expressing the Ascl1, Nurr1, and Lmx1a genes in naïve hPSCs (Theka et al., 2013).

As mentioned above, neurons derived from hPSCs require time to mature fully and are comparable to cells at the immature fetal stage (Marchetto and Gage, 2012). This is particularly troublesome when one aims to study the mechanisms of neuronal aging and associated neurodegenerative disorders. In fact, neuronal aging is a major contributor to late-onset neurodegenerative disorders and must be taken into account when modeling such disorders. In order to model late-onset diseases, recent studies have employed hPSC-derived neuronal *in vitro* cultures that have been grown for up to 200 days (Dimos et al., 2008; Ebert et al., 2009). hPSCs represent an interesting system with which to follow changes in the morphological and functional properties of neurons over a long period of time *in vitro*, possibly mimicking some of the processes that occur during aging. However, new solutions are needed to accelerate the aging process of these neurons *in vitro*. Notably, a new approach has been recently proposed that involves the expression of progerin, a truncated form of lamin-A that is associated with premature aging (Miller et al., 2013). In fact, the expression of progerin in hPSC-derived fibroblasts and neurons induced multiple aging-related markers and characteristics, including the appearance of neuromelanin in dopamine neurons (Miller et al., 2013). It remains to be established which features of late-onset disease can be authentically recapitulated by the progerin-induced model *in vitro*. Similar to this study, other known age-specific molecular pathways might be explored to promote neuronal aging *in vitro* (Newgard and Sharpless, 2013).

## Pathological neurogenesis: modeling human diseases using neurons generated *in vitro*

The advent of reprogramming adult human cells to become human induced pluripotent stem cells (hiPSCs) has revealed new opportunities to model disorders linked to a genetic cause. hiPSCs are able to self-renew and give rise to the affected somatic cells, providing an unlimited source of informative material to investigate diseases of interest. To date, a number of studies have validated this approach, modeling both neurological and neuropsychiatric disorders (Tiscornia et al., 2011; Zhu et al., 2011; Sandoe and Eggan, 2013). Monogenic diseases in which the mutated gene is known are particularly suitable for this approach. In this scenario, the function of the gene of interest can be restored, thus proving that the original mutation was indeed the causative factor of the disease-specific phenotype (Soldner et al., 2011; Liu et al., 2012). The approach is particularly relevant because the individual genetic variability of human sample often hampers any comparison between patient and healthy donor cells, disabling the detection of phenotype differences (Saha and Jaenisch, 2009). In fact, it would be necessary to analyze a large cohort of control and patient cells in parallel in order to address this matter, which would require a huge effort to generate and analyze a large range of hiPSC lines. Some compelling studies have been reported recently that illustrate the power of this approach in helping to identify new cellular and molecular mechanisms of disease; we report a few notable examples here.

Lee et al. (2010) developed an hiPSC model of familial dysautonomia, a disorder that causes depletion of the autonomic and sensory neurons, induced by mutations in the IKBKAP gene. In the hiPSC system, they were able to correlate the loss of IKBKAP gene expression with defects in neurogenesis and diminished cell motility in hiPSC-derived neural crest cells. Similarly, Marchetto et al. (2010) used neurons derived from the hiPSCs of individuals affected by Rett syndrome (RTT), an autism-like disorder caused by mutations in the methyl CpG binding protein MECP2. Lack of MeCP2 protein in hiPSC-derived neurons was associated with decreases in dendritic spines, synapses, and calcium oscillation frequency, as well as a concurrent reduction in nuclear size compared with control cells. A more recent study used an isogenic human embryonic stem cell model of RTT to identify a significant reduction in nascent protein synthesis (Li et al., 2013). Moreover, cells manifested a severe defect in AKT/mTOR pathway activity that resulted from the lack of MECP2 expression (Ricciardi et al., 2011; Li et al., 2013). More interestingly, the activation of AKT/mTOR signaling was sufficient to ameliorate the disease phenotypes in mutant hiPSC-derived neurons, by boosting protein synthesis. These data clearly show how the hiPSC modeling system is a convenient platform for the development of pharmacologic and genetic approaches that aim to revert a mutant phenotype using either a hypothesis-driven procedure or a more systematic solution.

A relevant question is whether hiPSC-based modeling can be fruitful for studying late-onset neurodegenerative diseases. In a recent study, hiPSC-derived neurons from patients with genetic or sporadic forms of Alzheimer disease (AD) did not exhibit enhanced susceptibility to cell death or changes in activity compared with controls (Israel et al., 2012). However, they did exhibit significantly higher levels of the pathological markers amyloid-β, phospho-Tau, and active GSK-3β than control cells. These hiPSC-derived neurons from AD patients also accumulated large RAB5-positive early endosomes compared with controls. Importantly, treatment of purified neurons with β-secretase inhibitors, but not γ-secretase inhibitors, caused significant reductions in phospho-Tau and GSK-3β levels (Israel et al., 2012). Therefore, a direct relationship can be drawn between the proteolytic processing of amyloid precursor protein, but not that of amyloid-β, in GSK-3β activation and Tau phosphorylation in hiPSC-derived neurons. These data suggest that hiPSC technology can be used to observe early pathological changes, possibly associated with the prodromal phase of AD, even if it does not faithfully recapitulate the phenotypes that characterize the late-onset phase.

## Neural induction from a single layer to three dimensions

The employment of cutting-edge culture procedures has recently elevated the potential of neural rosettes to recapitulate the events and mechanisms that underlie neural tube development to another dimension (Eiraku et al., 2008; Lancaster et al., 2013). Studies have recently reported the generation of *in vitro* 3D cell aggregates (organoids) that not only reproduce an organ's structure, but also recapitulate its function and development (Sato et al., 2009; Suga et al., 2011; Kadoshima et al., 2013). The applications of this technology to the investigation of still-unapproached aspects of organogenesis promise to bear relevant knowledge that is instrumental to a full understanding of tissue formation and pathogenesis.

The possibility of modeling human brain disease in a Petri dish (Eiraku et al., 2008) is certainly the most attractive, but innumerable implications can be imagined. Two different approaches have attempted to reproduce corticogenesis (and, even more surprisingly, brain morphogenesis) using hPSCs: quick re-aggregation of cells was determinant in the first case (Eiraku et al., 2008; Kadoshima et al., 2013), while larger and more complex structures (cerebral organoids) were achieved in the second case using a spinning bioreactor (Lancaster et al., 2013). Each approach generated a complex, multilayered structure that was able to self-organize and possessed morphogenetic features highly reminiscent of brain development. Among the most impressive: formation of an apical and basal membrane and specification of the anterior-posterior axis. Nonetheless, the specification of a larger number of brain areas (e.g., sub-pallial structures) was only detectable within cerebral organoids. This finding is particularly relevant because it has permitted the recapitulation of a key process of brain development that is highly correlated with numerous cortical deficits, such as the invasion of the neocortex by migrating GABAergic neurons originating in the sub-palladium.

Corticogenesis itself was also partially recapitulated in both cellular systems. Cortical development is a highly dynamic and complex process in which an internal layer of neuroepithelial progenitors (the ventricular zone) generates post-mitotic neurons that migrate and stratify in an inside-first outside-last fashion depending on their birth date (Molyneaux et al., 2007). As expected, the cardinal features of the neuroepithelial progenitors, already identified in neural rosettes, were also observed in the apical or luminal side of 3D structures, where progenitors reside. Further characterization of these cells revealed features typical of radial glial cells (RGCs, late progenitors of the neocortex), such as apical and basal processes. Moreover, a population of transient Tbr2-positive amplifying cells (which normally occupy the sub-ventricular zone of the developing cortex) was also identified adjacent to RGC-like cells. It is worth noting the description of a third type of progenitors predominantly derived from human cultures. The appearance of these cells (mainly characterized by the absence of the apical process) is highly reminiscent of outer-radial glial (oRG), an important hallmark of human neurogenesis in contrast to murine neurogenesis. The abundance of these cells in the developing human cortex has been associated with more complex structural features, such as cortical gyrification; thus, the presence of oRG-like cells indicates the faithful reproduction of human corticogenesis in 3D culture.

The identification of a progeny of post-mitotic neurons that form layers that are positioned basally over proliferative areas according to their time of birth has further confirmed the identity of the progenitor niche. The distribution and specification of post-mitotic neurons in quickly re-aggregated cells and in cerebral organoids has managed to recapitulate the early events of corticogenesis, although the realization of a fully layered cortex still remains to be established. Nonetheless, a simpler but equally laminated neuroepithelial structure, the retina (Eiraku et al., 2011; Nakano et al., 2012), was reproduced *in vitro* in similar culture conditions, once again confirming the ability of neuroepithelial cells to self-organize. The importance of this cardinal feature might lie in the ability of these cells to polarize (and consequently, to form rosettes). It was recently suggested that the same processes, polarization and rosette formation, are fundamentally involved in epiblast specification and self-organization, which emphasizes the intriguing hypothesis of a possible common mechanism in tissue morphogenesis.

The advent of 3D cultures can significantly change the way we see not only regenerative medicine, but also cell research en bloc. Although this system can provide unique information, great effort will have to be deployed to shepherd these expectations into solid reality.

### Conflict of interest statement

The authors declare that the research was conducted in the absence of any commercial or financial relationships that could be construed as a potential conflict of interest.
